# Transmission of light signals from the light-oxygen-voltage core via the hydrophobic region of the *β*-sheet surface in aureochrome-1

**DOI:** 10.1038/s41598-021-91497-5

**Published:** 2021-06-07

**Authors:** Hiroto Nakajima, Itsuki Kobayashi, Yumiko Adachi, Osamu Hisatomi

**Affiliations:** Department of Earth and Space Science, Graduate School of Science, Osaka University, Toyonaka, Osaka 560-0043 Japan

**Keywords:** Biophysics, Molecular biophysics

## Abstract

Light-Oxygen-Voltage (LOV) domains are responsible for detecting blue light (BL) and regulating the activities of effector domains in various organisms. Photozipper (PZ), an N-terminally truncated aureochrome-1 protein, contains a LOV domain and a basic leucin zipper (bZIP) domain and plays a role as a light-activatable transcription factor. PZ is monomeric in the dark state and undergoes non-covalent dimerization upon illumination with BL, subsequently increasing its affinity for the target DNA. To clarify the molecular mechanism of aureochromes, we prepared site-directed mutants of PZ and performed quantitative analyses in the dark and light states. Although the amino acid substitutions in the hinge region between the LOV core and A’*α* helix had minor effects on the dimerization and DNA-binding properties of PZ, the substitutions in the *β*-sheet region of the LOV core and in the A’*α* helix significantly affected these properties. We found that light signals are transmitted from the LOV core to the effector bZIP domain via the hydrophobic residues on the *β-*sheet. The light-induced conformational change possibly deforms the hydrophobic regions of the LOV core and induces the detachment of the A’*α* helix to expose the dimerization surface, likely activating the bZIP domain in a light-dependent manner.

## Introduction

Many organisms on earth use light as an information carrier and have evolved various photoreceptor molecules. The light-oxygen-voltage (LOV) domain is one of the photoreceptor domains found in various organisms (e.g., bacteria, fungi, algae, and plants)^1–3^. The LOV domain consists of ~ 100 amino acids and a chromophore represented by flavin mononucleotide (FMN). FMN in the LOV domain absorbs blue light (BL), forming a covalent adduct at the C4 position with a highly conserved cysteine residue and inducing protonation at the N5 position^4–6^. FMN subsequently induces the LOV core to undergo a conformational change to the light activated state. The cysteinyl adduct spontaneously dissociates, and the LOV domain returns to the dark ground state (dark regeneration). Most LOV domains are conjugated with effector domains that are generally located at the C-terminal of the LOV domain, and light information received by these domains is used for biological activities^1–3^. For example, plant phototropins have a kinase domain at the C-terminal, and BL induces the detachment of the Jα helix at the C-terminal of the LOV core, increasing the activity of the kinase domain^7–10^.

**Figure 1:**
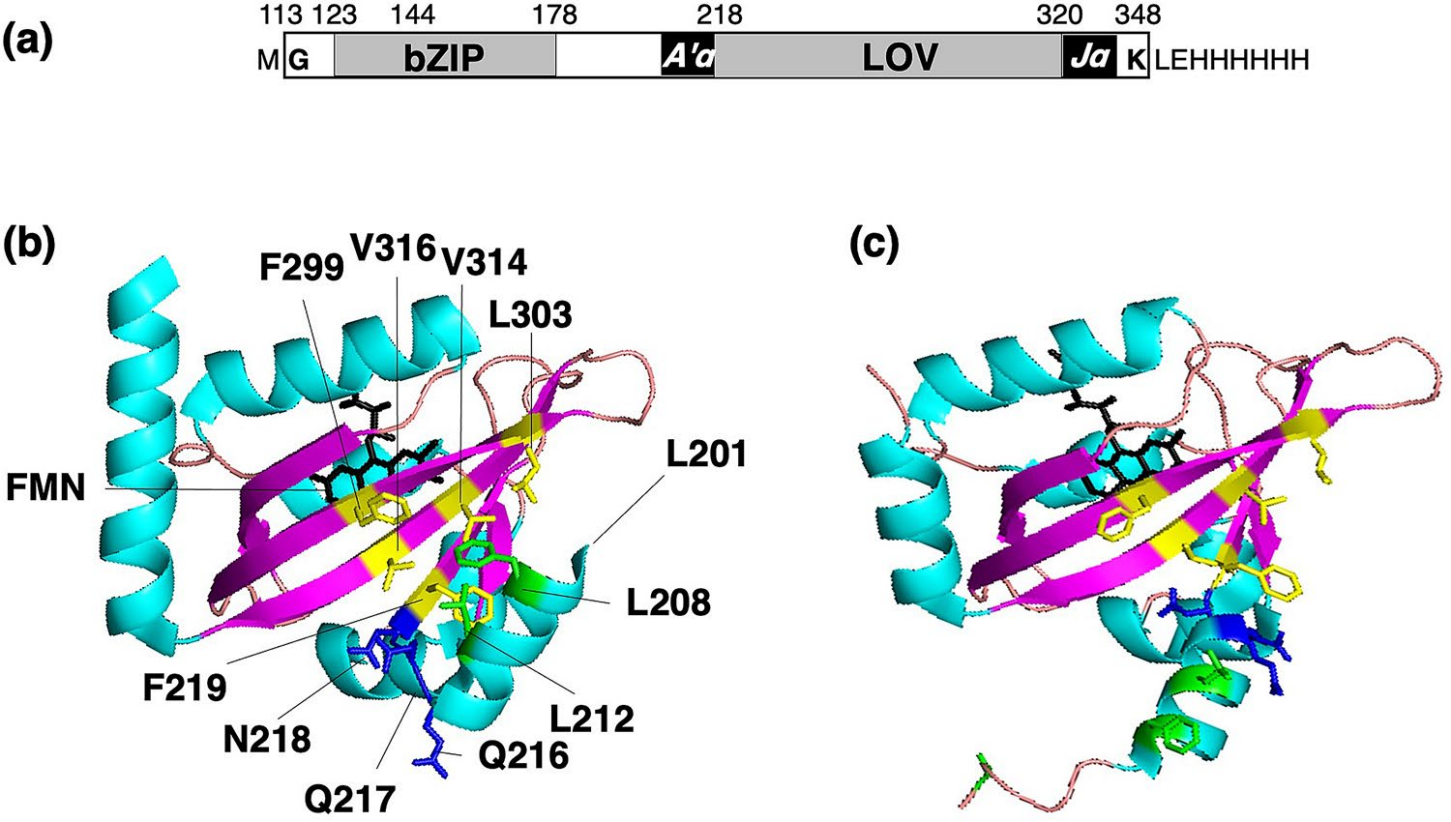
(**a**) The domain structure of PZ. Amino acids replaced in this study are indicated in the crystal structures of the *Pt*AUREO1a-LOV in the (**b**) dark and (**c**) light states (PDB: 5DKK and 5DKL). Amino acids in the *β*-sheet region, hinge region, and A’*α* helix are shown in yellow, blue, and green, respectively.

Aureochromes constitute a family of LOV domain-containing proteins (LOV proteins) found among stramenopiles^11,12^. They have a basic leucin zipper (bZIP) domain, which is an *α*-helical DNA-binding motif at the N-terminal of the LOV domain. In a previous study, aureochrome-1 of *Vaucheria frigida* (*Vf*AUREO1) was found to be responsible for the BL-induced branching response through transcriptional regulation^11^. We previously reported a synthetic gene (*op*ZL) encoding the N-terminally truncated *Vf*AUREO1 called “Photozipper” (PZ) (Fig. 1a)^13^. PZ, which contains bZIP and LOV domains, shows an absorption band characteristic of LOV domains with an absorption maximum (*λ*_max_) at 447 nm. Dynamic light scattering (DLS) and size-exclusion chromatography (SEC) have been used to indicate that PZ exists as a monomer in the dark state, and BL induces the non-covalent dimerization of PZ^13,14^. We previously developed the quantitative measurements to evaluate the affinity of PZ for target DNA sequences through electrophoretic mobility shift assay (EMSA) and quartz crystal microbalance (QCM), and clarified that PZ increases its affinity for the target DNA upon illumination^15–17^. To elucidate the intramolecular signaling mechanism, we quantitatively measured the monomer–dimer equilibrium and DNA binding of PZ mutants with amino acid substitutions of F298 and Q317^17^. Since all mutants increased the dimer fractions and affinities for DNA upon illumination, we concluded that there is an equilibrium between “closed” and “open” conformations of the LOV domain and suggested that the BL-regulated switching of *Vf*AUREO1 is achieved by the shift in this equilibrium driven by a synergistic interaction between the chromophore and protein moiety. However, the LOV core signaling pathway that activates the bZIP domain is still elusive.

Mitra et al. reported the tertiary structure of *Vf*AUREO1-LOV by analyzing the crystal prepared in the dark and suggested that F298 of the *β*-sheet displays distinct side-chain conformations in the dark and light states^18^. Heintz and Schlichting successfully produced the crystals of the aureochrome-1a of the diatom *Phaeodactylum tricornutum* (*Pt*AUREO1a) LOV domain under both dark and light conditions and proposed a model in which BL induces the undocking of J*α* from the *β*-sheet of the LOV core and releases the A’*α* helix from the hydrophobic dimerization site^19^. In contrast, Kalvaitis et al. recently suggested from their studies on the AUREO1a-LOV of *Ochromonas danica (Od*AUREO1a-LOV) that the formation of a new hydrogen bond network creates a route for the conformation of Gln293 to be communicated to the A’*α* helix through Asn194 (corresponding to N218 of *Vf*AUREO1)^20^. Therefore, two hypothetic LOV core-signaling mechanisms that activate the bZIP domain have been proposed: (1) reconstruction of a hydrogen bond network in the hinge region between the LOV core and A’*α* helix; and (2) deformation of the hydrophobic region on the *β*-sheet of the LOV core.

There are hydrophobic regions on the *β*-sheet of the LOV core in most LOV domains. However, the roles of these hydrophobic regions remain unknown, probably because hydrophobic interactions are more delicate than hydrogen bonds. In this study, we prepared site-directed mutants, in which amino acids conserved among aureochromes were substituted for smaller residues (Ala and Val), and performed quantitative evaluations of dimer formation and DNA binding. Amino acid substitutions are in the hinge region between the A’*α* helix and LOV core (hinge mutants: Q216A, Q217A, and N218A), in the *β*-sheet of the LOV core (*β*-sheet mutants: F219V, F299V, L303A, V314A, V314I, and V316A), and in the A’*α* helix (A’*α* mutants: L201A, L208A, L212A, and a triple mutant L201A/L208A/L212A) (Fig. 1b,c).

## Methods

### Preparation of recombinant proteins

To prepare the site-directed mutants of PZ, the expression vector containing the *op*ZL gene encoding G113–K348 of *Vf*AUREO1 with C162S and C182S substitutions (Fig. 1a)^13^ was mutated using a PrimeSTAR mutagenesis kit (Takara Bio, Shiga, Japan) and primer sets: Q216A-F and Q216A-R, Q217A-F and Q217A-R, N218A-F and N218A-R, F219V-F and F219V-R, F299V-F and F299V-R, L303A-F and L303A-R, V314A-F and V314A-R, V314I-F and V314I-R, and V316A-F and V316-R (Supplementary Table S1). Four sets of primers, L201A-F and L201A-R, L208A-F and L208A-R, L212A-F and L212A-R, and L212A-F and L208A/L212A-R, were used to prepare the A’*α* mutants. Recombinant PZ mutants were isolated as previously reported^14,21,22^.

### Spectroscopic measurements

Recombinant proteins were diluted to 4 µM in a loading buffer (400 mM NaCl, 20 mM Tris–HCl, pH 7.0) containing 1 mM DTT. UV–visible absorption spectra were measured using a V550 spectrophotometer (JASCO, Tokyo, Japan) as previously reported^22^. Spectral changes accompanying dark regeneration were monitored at 25 °C, and the regeneration curves were calculated using the maximum absorbance difference (∆*A*_*max*_) at the *λ*_*max*_ of each mutant. Each regeneration curve was fitted to a single exponential formula, ∆*A*_max_ (1-e^-*kt*^), where *k* is the rate constant of dark regeneration.

### DLS

DLS of the protein solutions were measured using a Zetasizer µV system (Malvern Panalytical, Malvern, UK) in automatic mode at 25 °C, and the z-average molecular sizes expressed as the apparent hydrodynamic radii (*R*_*H(app)*_) in solution were determined using Zetasizer software (version 7.12) as previously described^14,16^. After removing the aggregates by ultra-centrifugation, DLS analyses of the recombinant proteins (30–60 µM) were conducted several times in the dark (D state), immediately after the termination of BL illumination for 1 min (L state), and after regeneration in the dark (LD state). *R*_*H (app)*_ of the samples was plotted against the concentration, and hydrodynamic radii (*R*_*H*_) values were obtained from the extrapolated data at 0 µM protein concentration^23^.

### SEC

SEC was performed using an ÄKTA purifier column chromatography system (Cytiva, Marlborough, MA, USA) with a Superdex 75 30/10 column in a loading buffer at a temperature of 25 ± 1 °C and flow rate of 0.5 mL/min^13,14,17^. To analyze proteins in the D state, reaction mixtures (containing 8 or 50 µM recombinant proteins) were incubated for 20 min at 25 °C in the dark, and 200-µL aliquots were subjected to SEC. To analyze proteins in the L state, reaction mixtures were illuminated for 2 min with BL and applied to the column under continuous irradiation with an LED. The molecular weight (MW) was calculated from elution peak volumes using the following formula^17,23^:

1 $$MW=104\times exp(-6.4\times {K}_{av})$$

### EMSA

Alexa Fluor647-labeled double-stranded palindromic oligonucleotides with (dsApo) or without (dsCpo) the AUREO1 target sequence (Supplementary Table S1) were incubated with various concentrations of PZ mutants, and each reaction mixture was separated in a 5% polyacrylamide gel at 25 ± 1 °C^15–17^. To investigate DNA binding in the L state, each reaction mixture was irradiated with BL and separated by electrophoresis under gel illumination with BL (5 W/m^2^). The fluorescence signal from the Alexa Fluor647-labeled oligonucleotide of each DNA band was quantified using a Fluor-S MAX image analyzer (Bio-Rad, Hercules, CA, USA) and ImageJ software^24^. The fraction of dsApo bound to PZ mutants was determined from the ratio of free dsApo to total dsApo, normalized by the amount of free ssApo, and plotted against the monomer concentrations of PZ mutants applied to the mixture ([*monomer*]_0_). *EC*_*50*_ was estimated by curve fitting to the Hill equation using the maximum of the bound fraction (*B*_*max*_).

### QCM

The DNA binding of each PZ mutant was detected using a 27 MHz-QCM system (Single-Q, AS ONE, Osaka, Japan) as described previously^16,17^. Briefly, biotinylated dsApo was anchored on the Au electrode of the QCM sensor, and the frequency change of the electrode was measured in the QCM buffer with a stirring rate of 120 RPM at 25 °C. In the D state, 10 µL of protein solutions (1–5 µM) were added to the solutions in the chamber for each injection. In the L state, protein solutions were irradiated for 2 min with BL and applied to the solutions in the chamber while being continuously illuminated with a blue LED. The frequency change due to the DNA binding to PZ mutants was plotted against [*monomer*]_0_. In each case, *EC*_*50*_ was estimated by curve fitting to the Hill equation using the maximum of the frequency change (*∆F*_*max*_).

## Results

### Spectroscopic properties

**Figure 2:**
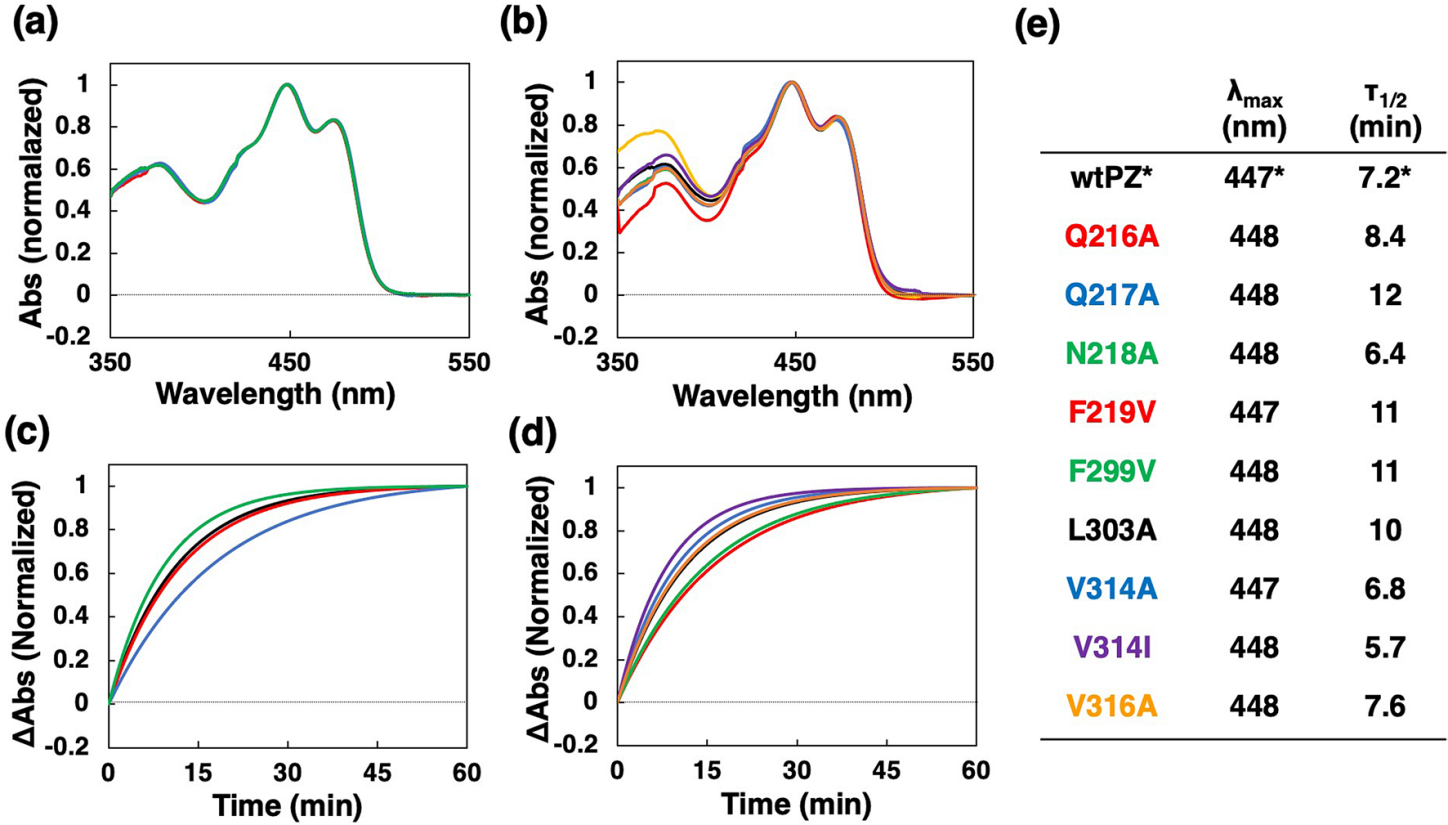
Normalized absorption spectra of (**a**) wtPZ and hinge mutants and (**b**) *β*-sheet mutants. Dark regeneration time courses measured at *λ*_*max*_ for (**c**) wtPZ and hinge mutants and (**d**) *β*-sheet mutants. (**e**) *λ*_*max*_ and *τ*_*1/2*_ values of dark regeneration. *Data for wtPZ were taken from a previous study^16^.

We initially prepared nine site-directed PZ mutants with amino acids substitutions in the hinge region (hinge mutants: Q216A, Q217A, and N218A) and in the *β-*sheet of the LOV core (*β*-sheet mutants: F219V, F299V, L303A, V314A, V314I, and V316A). The absorption spectra in the D state were almost identical to that of wild-type PZ (wtPZ), showing triplet vibrational structures characteristic of LOV domains (Fig. 2a,b)^22^. Moreover, the absorption maxima were detected at 447 or 448 nm (Fig. 2e), indicating that all mutants accommodate FMN precisely in the same binding site. The half-reaction times (*τ*_*1/2*_) of dark regeneration were distributed from 5.7 to 12 min (Fig. 2c–e), but mutant PZs showed similar spectral changes immediately after BL illumination (Supplementary Fig. S1, dashed lines), and during dark regeneration (Supplementary Fig. S1, gray lines). Although mutations may cause the subtle difference in the dark regeneration kinetics, amino acid substitutions have a minor effect on the spectroscopic properties and photoreaction of PZ.

### DLS

Since *R*_*H(app)*_ measured by DLS depends on the protein concentration^14,23^, the *R*_*H(app)*_ values were plotted against the monomer concentration subjected to each measurement ([*monomer*]_0_) in the dark (D state, black symbols and lines), immediately after BL illumination (L state, blue symbols and lines), and after dark regeneration (LD state, green symbols and lines) (Supplementary Fig. S2). The *R*_*H*_ values of PZ mutants were evaluated by extrapolating protein concentration to zero (Table 1). The *R*_*H*_ values of the hinge mutants were similar to those of wtPZ in both the D and L states. On the other hand, *β*-sheet mutants showed dispersed *R*_*H*_ values and can be classified into three groups: (1) mutants with *R*_*H*_ of ~ 3.7 nm irrespective of light conditions (F219V and V314I); (2) mutants with similar *R*_*H*_ in the D state but smaller *R*_*H*_ (~ 3.4 nm) in the L state (F299V and L303A); and (3) mutants with greater *R*_*H*_ (~ 3.2 nm) in the D state but similar *R*_*H*_ (~ 3.8 nm) in the L state (V314A and V316A).

**Table 1:** *R*_*H*_ of wtPZ and PZ mutants.

	D (nm)	L (nm)	LD (nm)
wtPZ	2.94*	3.75*	2.96*
Q216A	2.95	*3.78*	3.10
Q217A	2.87	*3.59*	2.91
N218A	2.92	*3.66*	3.04
F219V	3.64	*3.84*	3.61
F299V	2.81	*3.42*	2.83
L303A	2.80	*3.37*	2.78
V314A	3.28	*3.80*	3.30
V314I	3.61	*3.61*	3.57
V316A	3.16	*3.73*	3.14

*Data for wtPZ were taken from a previous study^16^.

### SEC

**Figure 3:**
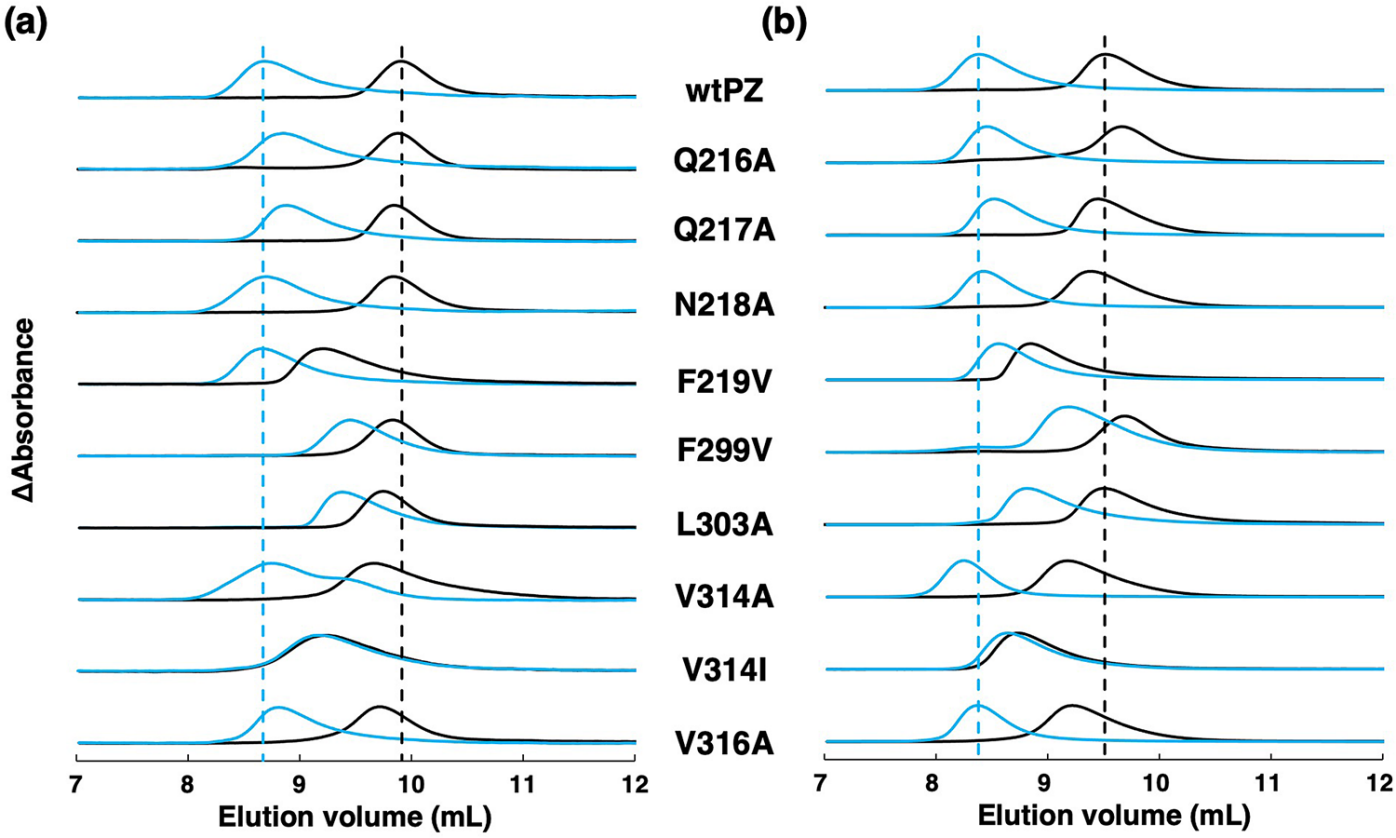
Elution profiles of wtPZ and PZ mutants monitored at 280 nm and at injected protein concentration of (**a**) 8 µM or (**b**) 50 µM. Black and blue lines indicate the profiles normalized to the maximum absorbance in the D and L states, respectively. Black and blue dashed vertical lines indicate the peak volumes of wtPZ in the D and L states, respectively.

The sizes of PZ mutants were further investigated by SEC at 8 µM (Fig. 3a) and 50 µM concentrations (Fig. 3b). The elution peak volumes of PZ mutants were found between 8.8 mL and 9.9 mL in the D state and between 8.2 and 9.4 mL in the L state (Supplementary Table S2). The concentration dependence of the elution peak volumes suggests that the dispersion of peak volumes depends primarily on the changes in monomer and dimer fractions rather than the structural variation of each mutant. Moreover, the peak volumes showed clear correlations with the *R*_*H*_^3^ value of each mutant at both 8 and 50 µM concentrations (Supplementary Fig. S3a,b). These findings are similar to those for the F298 and Q317 mutants^17^. The correlation between the D and L states at the concentration of 50 µM appeared to be superior to that at 8 µM, probably because 50 µM approximates the concentrations (30–60 µM) under the DLS conditions. Therefore, we conclude that the variation of *R*_*H*_ and peak volumes of PZ mutants are caused by the shift in the monomer–dimer equilibrium.

The peak volumes of the Q216A and Q217A mutants showed minimal right-shifts compared to that of wtPZ in the L state, suggesting that the substitutions slightly destabilize the dimer form. However, the peak volumes of the N218A mutant were similar to those of wtPZ in both the D and L states, clearly indicating that the N218A substitution has a minor effect on the monomer–dimer equilibrium of PZ. On the other hand, the elution profiles of the *β*-sheet mutants showed significant variation. The peak volume of the F219V mutant in the D state was significantly left-shifted but similar to that in the L state, indicating that most of F219V is dimeric irrespective of the light conditions. Likewise, the V314I mutant showed elution profiles that were similar in the D and L states, suggesting that the substitution of V314 for a larger residue (Ile) completely suppresses BL-induced dimerization. The peak volumes of the F299V and L303A mutants were significantly greater in the L state than in the D state, indicating that F299V and L303A substitutions decrease dimer formation in the L state. The elution profiles of the V314A and V316A mutants suggest that the substitutions involved slightly induce the destabilization of monomer and dimer forms in the D and L states, respectively. At 50 µM, the peak volume of the V314A mutant appeared to be smaller than that of wtPZ in the L state, probably due to the oligomerization of this mutant at high concentrations. The elution peak volume of each PZ mutant is consistent with the *R*_*H*_ value of each mutant obtained by DLS measurements.

### Affinities for DNA

The affinities of PZ mutants for target and non-target sequences were quantitatively analyzed by EMSA. Supplementary Fig. S4 shows the fractions of dsApo bound to each PZ mutant plotted against the protein concentration [*monomer*]_0_. The maximum of the bound fraction (*B*_*max*_, where [*monomer*]_0_ = ∞) and *EC*_*50*_ were estimated by fitting the data to the Hill equation. The specificities of DNA binding were confirmed using the control dsCpo (Supplementary Fig. S5).

**Figure 4:**
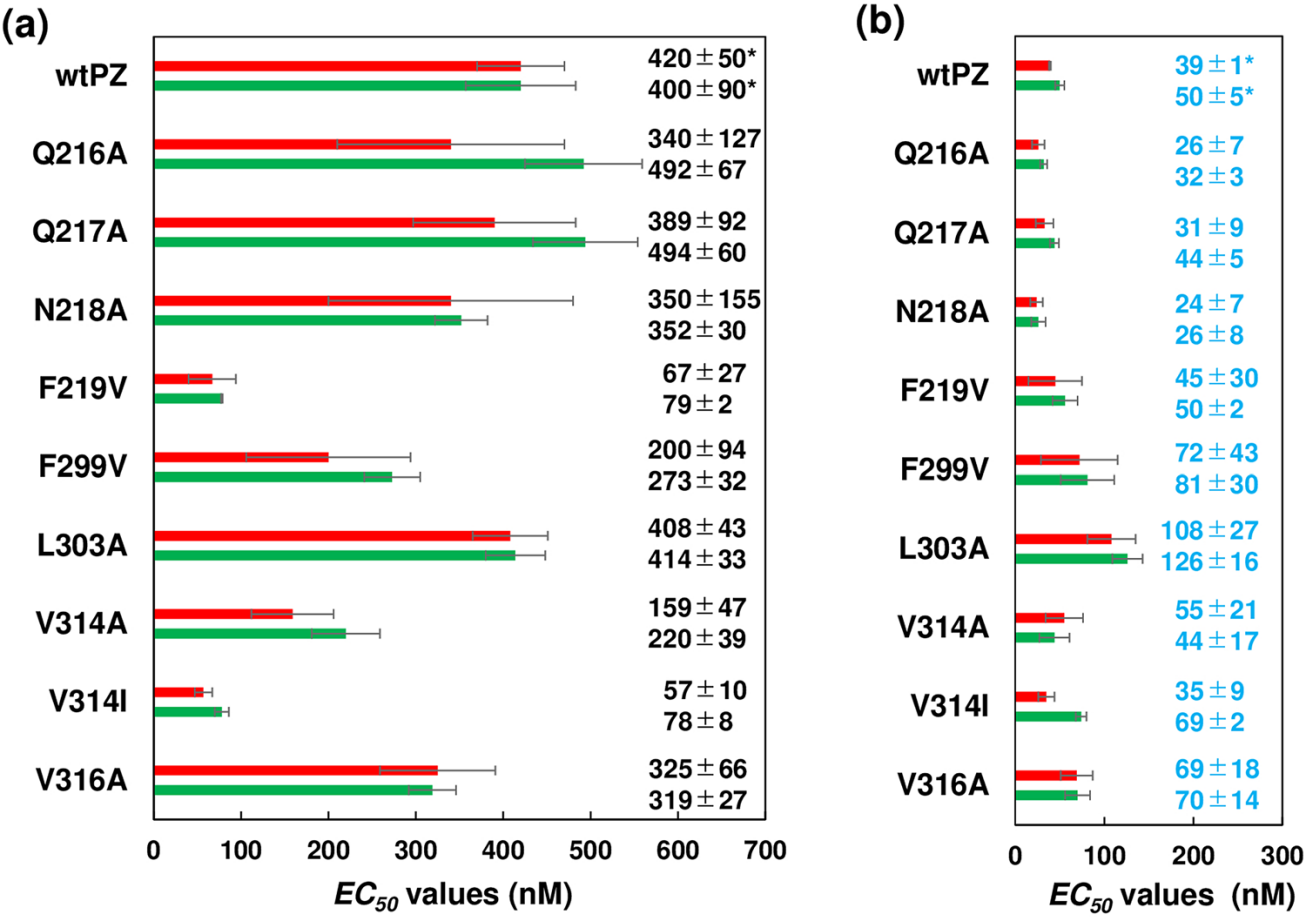
The *EC*_50_ values of wtPZ and PZ mutants for dsApo binding according to EMSA (red bars) and QCM (green bars) in the (**a**) D and (**b**) L states with error bars (n ≥ 3). Numbers indicate *EC*_50_ values and standard deviations (nM). *Data for wtPZ were taken from previous studies^15,16^.

The affinity of each PZ mutant for dsApo was also measured by QCM. With the injection of PZ mutants, the resonance frequency of the electrode decreased toward the frequency in equilibrium (*∆F*_*eq*_) due to the formation of the PZ-dsApo complex. Supplementary Figure S6 shows the plot of *∆F*_*eq*_/*∆F*_*max*_ against [*monomer*]_0_ of each PZ mutant. The *EC*_*50*_ values of each PZ mutant estimated using EMSA and QCM showed good agreement with each other in the D (Fig. 4a) and L states (Fig. 4b), demonstrating that our DNA-binding assays are quantitatively credible. The hinge mutants and wtPZ showed similar *EC*_*50*_ values in both the D and L states. The F299V and L303A mutants exhibited high *EC*_*50*_ values in the L state, as shown in both DLS and SEC data, while the F219V mutant showed low *EC*_*50*_ values even in the D state. The *EC*_*50*_ values of the V314I mutant were similar in the D and L states, indicating that the substitution of V314 for Ile suppresses the BL-induced activation of the effector bZIP domain.

### Substitutions of Leu residues in the A’*α* helix

**Figure 5:**
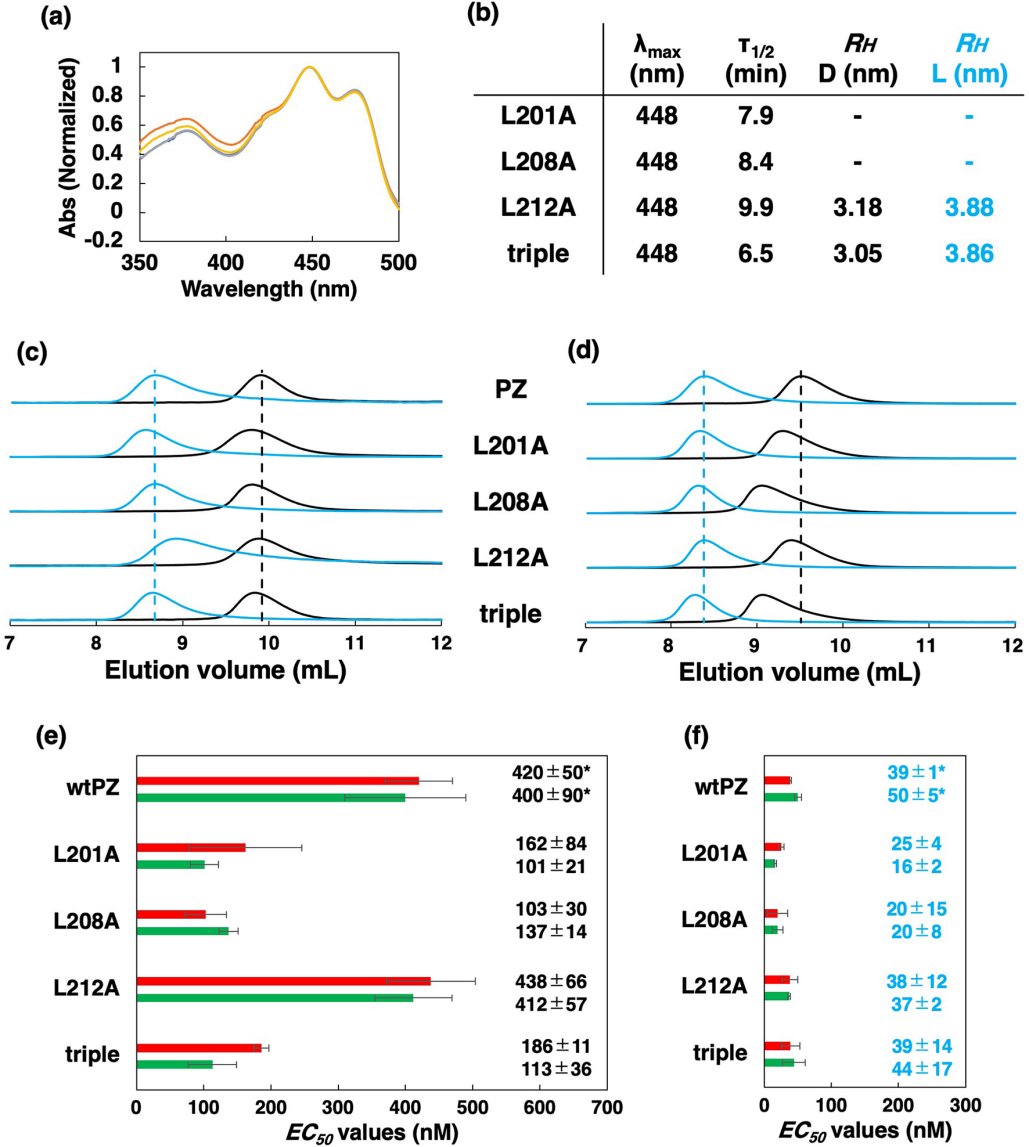
(**a**) Normalized absorption spectra of the A’α mutants. (**b**) *λ*_*max*_ in the D state and *τ*_*1/2*_ values of dark regeneration with *R*_*H*_ values of the L212A and triple mutant in the D and L states. Elution profiles of the A’α mutants in the D (black lines) and L (blue lines) states, monitored at 280 nm at injected protein concentration of (**c**) 8 µM or (**d**) 50 µM. Black and blue dashed vertical lines indicate the peak volumes of wtPZ in the D and L states, respectively. The *EC*_*50*_ values of the A’α mutants for dsApo according to EMSA (red bars) and QCM (green bars) in the (**e**) D and (**f**) L states with error bars (n ≥ 3). Numbers indicate *EC*_50_ values and standard deviations (nM). *Data for wtPZ were taken from previous studies^15,16^.

Our data strongly suggest that light information is transmitted via the hydrophobic region of the *β*-sheet from the LOV core. Thus, we prepared four mutants with amino acid substitutions in the A'*α* helix (L201A, L208A, L212A and a triple mutant L201A/L208A/L212A), assuming that these Leu residues interact with F219, V314, and V316 on the *β*-sheet. The absorption spectra and dark regeneration kinetics of the A'*α* mutants were similar to those of wtPZ (Fig. 5a,b, Supplementary Fig. S7). Although we failed to determine the accurate *R*_*H*_ of L201A and L208L mutants due to their aggregation tendency, L212A had similar *R*_*H*_ with the V314A and V316A mutants in both the D and L states (Fig. 5b). SEC measurements indicated that the peak volumes of L201A, L208A and the triple mutant were left-shifted in comparison with those of wtPZ in the D state (Fig. 5c,d, Supplementary Table S3). Similar shifts were detected for V314A and V316A mutants, suggesting that L201 and L208 stabilize the monomeric form in the D state. The *EC*_50_ values of L212A was similar to those of wtPZ both in the D and L states (Supplementary Figs. S8 and S9, Fig. 5e,f), suggesting that L212 has a minor effect on the conformational stability of PZ. On the other hand, the *EC*_50_ values of L201A, L208A and the triple mutant for the target DNA binding were lower than that for wtPZ in the D state, which is consistent with the SEC data that Leucine residues at 201 and 208 positions stabilize the monomeric form in the D state. Although small *EC*_50_ values of L201A and L208A mutants in the L state are possibly due to their aggregation tendency, our data are consistent with the assumption that L201 and L208 in the A'*α* helix primarily interact with the hydrophobic residues of the *β*-sheet in the LOV core and stabilize the monomeric form in the D state.

## Discussion

**Figure 6:**
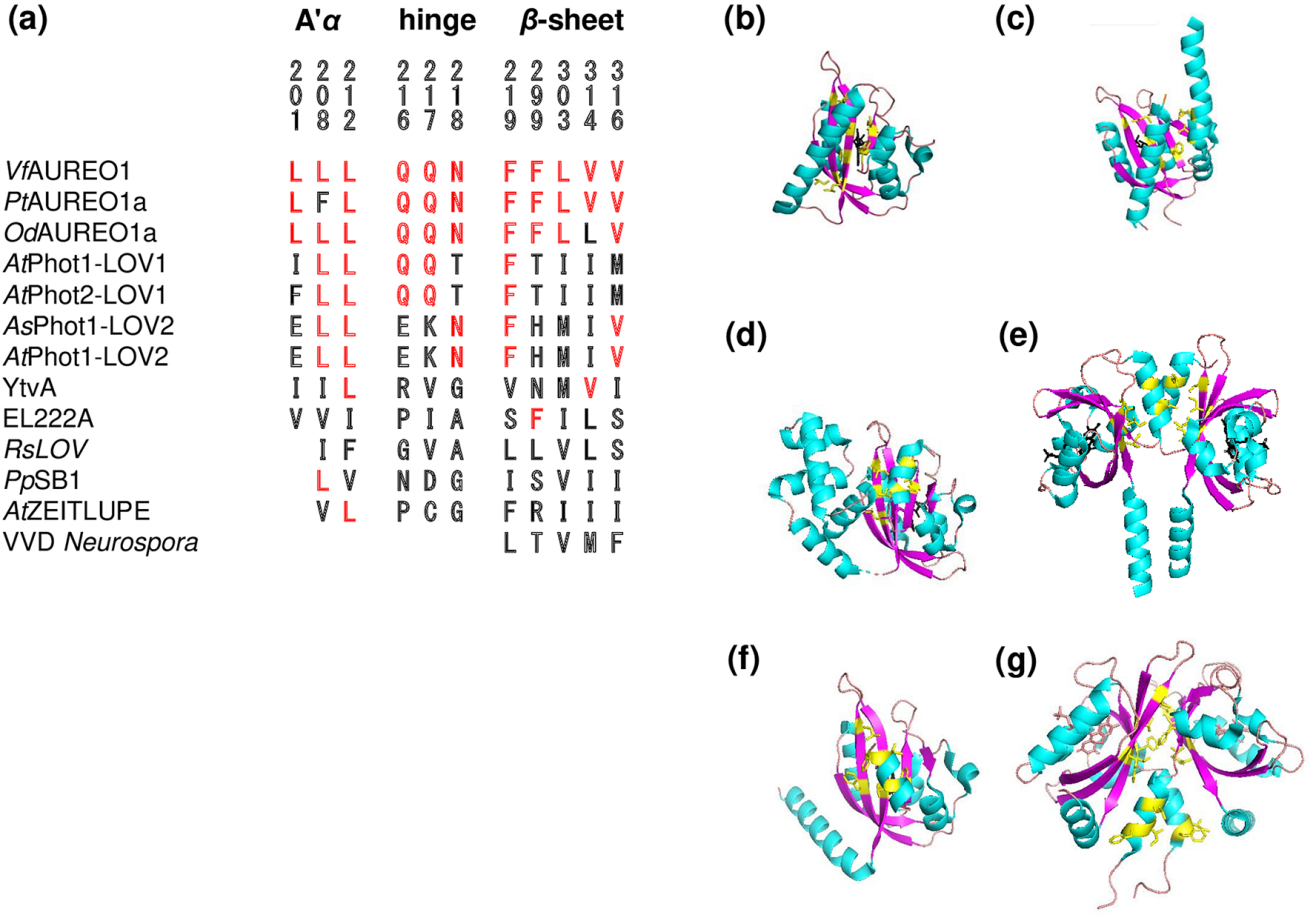
(**a**) The amino acids of *Vf*AUREO1 investigated in this study, aligned with those of other LOV proteins. Amino acids shown in red represent residues identical to those of *Vf*AUREO1. Structures of (**b**) *As*LOV2 (PDB:2V0U), (**c**) *Pt*Phot1-LOV2 (PDB:4HHD), (**d**) EL222 (PDB:3P7L), (**e**) PpSB1 (PDB:5J4E), and *Vf*AUREO1 in the (**f**) dark (PDB:5DKK) and (**g**) light (PDB:5DKL) states. Amino acids shown in yellow correspond to the hydrophobic residues in the *β*-sheet and A’*α* helix.

The hydrophobic amino acids (L201, L208, L212, F219, F299, L303, V314, and V316 of *Vf*AUREO1) in the A'*α* helix and *β*-sheet are well conserved among the LOV domains of aureochromes (Fig. 6a). In LOV1 and LOV2 of plant phototropins, L208, L212, and F219 are highly conserved, but Ile is located at the position corresponding to V314. The V314I mutation resulted in acquisition of constitutive activity for the signal transduction ability of PZ; such occurrence may be related to the signal transduction of phototropins toward the C-terminal. Amino acids corresponding to F299, L303, and V316 are Thr, Ile, and Met, respectively, in phototropin-LOV1 and His, Met, and Val, respectively, in phototropin-LOV2. These residues may be responsible in producing the characteristic difference between LOV1 and LOV2 in plant phototropins. The hydrophobic residues in the *β*-sheet regions are also found in many other LOV domains. The structural analyses suggest that the hydrophobic residues participate in the formation of either an intramolecular hydrophobic region with J*α* and/or N-terminal helices or an intermolecular hydrophobic region with a dimer pair. The hydrophobic residues in phototropin-LOV2 are covered by the J*α* and A’*α* helices (Fig. 6b,c)^25,26^. Similarly, these residues are sequestered by *α*-helices of the same molecule in *Erythrobacter* EL222, *Neurospora* VVD, and *Rhodobacter sphaerioides* LOV (*Rs*LOV) (Fig. 6d)^27–30^. In contrast, the hydrophobic residues in the *β*-sheet of *Bacillus* YtvA and *Pseudomonas putida* LOV (*Pp*SB1) are located at the hydrophobic interface of a dimer (Fig. 6e)^31–33^. The structural diversity among the LOV domains is likely due to the interactions on the hydrophobic *β*-sheet surface of the LOV core.

Molecular mechanisms that involve the regulation of effector activity by LOV domains in phototropins have been studied. Harper et al. produced a series of point mutations along the J*α* helix to disrupt the interaction between the helix and the LOV core and demonstrated that several of these mutations displayed constitutive kinase activation in the absence of illumination^8^. Jones et al. reported that the I608E mutation within the J*α* helix resulted in the activation of phot1 autophosphorylation in the absence of light^9^. In addition, dissociations of the N-terminal region from the LOV core have been reported for *Avena sativa* LOV2 (*As*LOV2) and *Arabidopsis* phototropin1 LOV2 (*At*phot1LOV2)^25,34,35^. For aureochromes, the crystal structures of *Pt*AUREO1a-LOV indicated that the A'*α* helix is in contact with the hydrophobic surface of the *β*-sheet in the dark state but is detached from the LOV core in the L state (Fig. 6f,g)^19^. Spectroscopic studies have suggested that BL induces the unfolding of both J*α* and A'*α* helices of *Vf*AUREO1-LOV and *Pt*AUREO1a-LOV^36,37^. The detachment of the A’*α* helix likely activates (or is synchronized with the activation of) the bZIP domain in aureochromes.

Through the structural analysis of *Od*AUREO1a-LOV, Kalvaitis et al. proposed a model in which the rotation of the side chain of Asn194 (corresponding to N218 of *Vf*AUREO1) due to a rearrangement of hydrogen bonds leads to conformational changes in the A'*α* helix^20^. Through simulation analyses, Tian et al. reported that A248, Q249, Q250, and N251 in the hinge region are the residues important for signal transduction in *Pt*AUREO1a^38^. The rearrangement of hydrogen bonds in the N-terminal region was also suggested for Neurospora VVD^27^. Zoltovskii and Crane surmised that the flavin protonation state is influenced by a series of compensatory conformational and hydrogen bonding changes from Q182 and C71 to Ncap^39^. In another simulation study, Gangly et al. also suggested that the hinge-b*β* is directly affected by the new hydrogen bond to Ala72 upon Gln182 rearrangement^40^. However, our data clearly demonstrates that amino acid substitutions in the hinge region have minor effects on the dimer formation and DNA binding of PZ. In *Vf*AUREO1, the displacement of the hinge region unlikely drives the detachment of the A'*α* helix upon BL-illumination.

Although hydrophobic residues are often found in the *β*-sheet of the LOV core, the roles of each residue remain to be clarified, probably because hydrophobic interactions are not that simple to investigate compared with hydrogen bonds. In the present study, we produced a series of PZ mutants in which the hydrophobic amino acids in the *β*-sheet were substituted for smaller residues and quantitatively measured the monomer–dimer equilibria and DNA-binding activities. Substitutions of F299 and L303 for smaller residues appeared to destabilize the dimer form in the L state; such finding is consistent with structural data showing that the F299 and L303 residues are located beside the A*’α* helix in the D state and at the peripheral region of the dimerization surface in the L state^19^. When V314 was replaced by a larger residue (Ile), the mutant forms dimer and binds DNA irrespective of light irradiation, probably because the V314I substitution caused a large deformation of the hydrophobic regions in both the D and L states. In contrast, the F219V, V314A, and V316A mutants, similar to the L201A and L208A mutants, exhibited altered *R*_*H*_ and *EC*_*50*_ values in the D state, suggesting that the substitutions involved in these mutants induce the partial detachment of the A’*α* helix in the D state.

**Figure 7:**
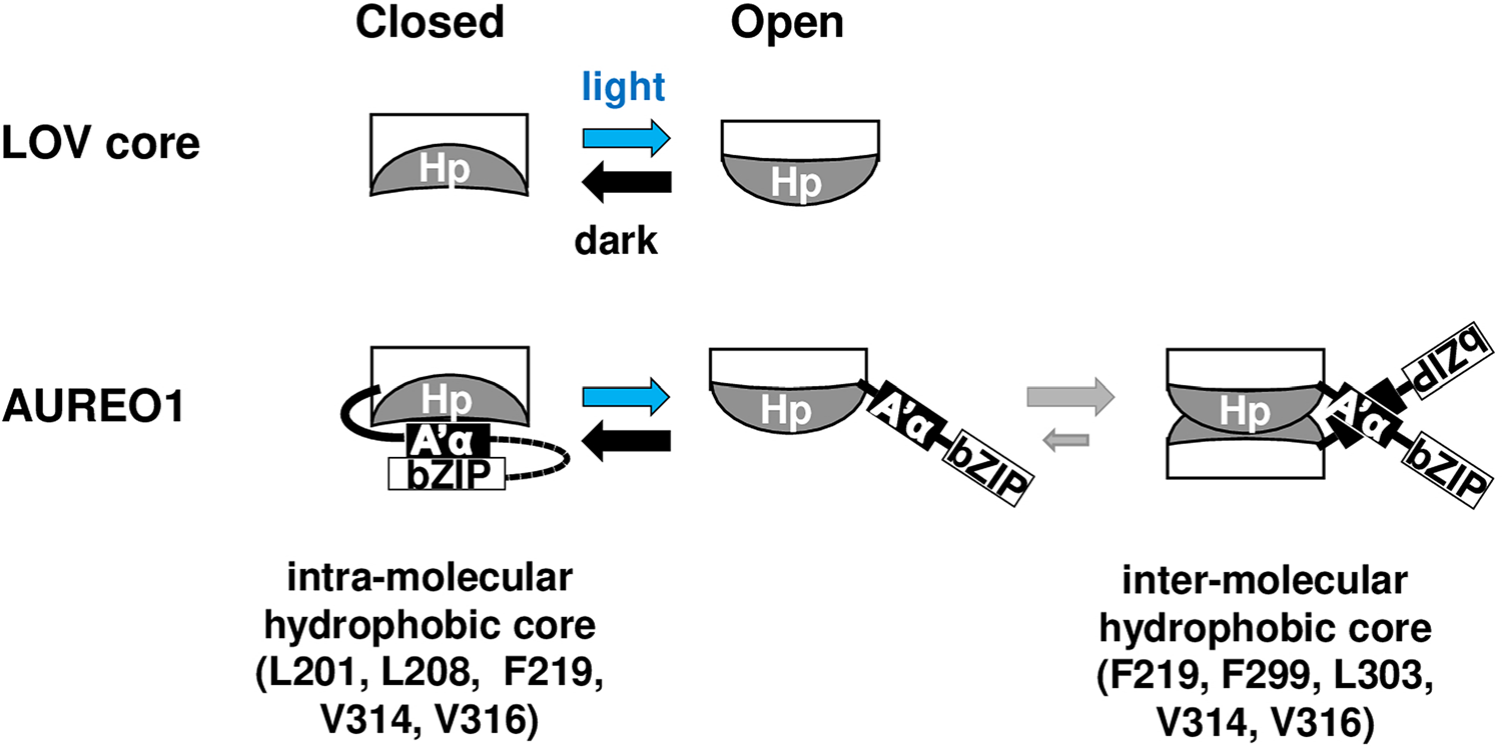
The closed-open conformational equilibrium of the LOV core (top). The equilibrium is shifted toward the closed conformation in the dark state and toward the open conformation in the presence of BL. Hp indicates the hydrophobic region in the *β*-sheet. In AUREO1 (bottom), the closed conformation is stabilized by intramolecular hydrophobic interactions involving L201, L208, and L212 in the A'*α* helix and F219, V314, and V316 in the *β*-sheet. BL shifts the equilibrium of the LOV core toward the open conformation and deforms the hydrophobic surface of the *β*-sheet containing F219, V314, and V316, subsequently inducing the detachment of the A'*α* helix and the dimerization of AUREO1. The AUREO1 dimer is stabilized by intermolecular hydrophobic interactions involving F219, F299, L303, V314, and V316.

We previously reported the correlation between the *R*_*H*_ and *EC*_*50*_ values of the F298 and Q317 mutants, due to the existence of the closed-open conformational equilibrium of the LOV core that regulates the monomer–dimer equilibrium of PZ^17^. In the present study, the correlations between the *R*_*H*_^3^ and *EC*_*50*_ values of the hinge and *β*-sheet mutants were similar to those of the F298 and Q317 mutants (Supplementary Fig. S10), strongly suggesting that the BL-induced shift in the conformational equilibrium of the LOV core is prompted by the deformation of the hydrophobic region of the *β*-sheet and induces the detachment of the A*’α* helix to expose the dimerization surface (Fig. 7). In the D state, the closed-open equilibrium of the LOV core shifts toward the closed conformation, and F219, V314, and V316 form the intramolecular hydrophobic region with L201 and L208 in the A'*α* helix, stabilizing the monomeric form. In the L state, the shift in the conformational equilibrium induces the detachment of the A*’α* helix to expose the dimerization surface composed of F219, F299, L303, V314, and V316, stabilizing dimer formation and resulting in the increased affinity of PZ for the target DNA sequence. The deformation of the hydrophobic regions in the *β*-sheet may be a general mechanism for signal transduction in the LOV domains that lack the BL-induced rearrangement of a hydrogen bonding network.

## Conclusion

To elucidate the molecular mechanism of the LOV domain in aureochromes, we prepared site-directed mutants of PZ. Amino acid substitutions in the hinge region between the A’*α* helix and the LOV core, and in the hydrophobic regions of the *β*-sheet have minor effects on the spectroscopic properties of PZ. Although the BL-induced dimerization and DNA binding properties of the hinge mutants were similar to those of wtPZ, those of the *β*-sheet mutants were significantly different, indicating that light signals are transmitted via the hydrophobic residues in the *β*-sheet. Moreover, the substitutions of Leu201 and Leu208 for Ala in the A*’α* helix affected the dimerization and DNA binding similar to those in the *β*-sheet mutants, suggesting that the BL-induced deformation of the hydrophobic regions causes the detachment of the A*’α* helix and results in the activation of the effector bZIP domain in aureochrome-1. The deformation of the hydrophobic regions may be a general mechanism for the transmission of light signals in the LOV domains that lack the BL-induced rearrangement of a hydrogen bonding network.

## Supplementary Information

Supplementary Information.
