# Supplementary material for: Transmission of light signals from the light-oxygen-voltage core via the hydrophobic region of the β-sheet surface in aureochrome-1
Source: Sci Rep. 2021 Jun 7;11:11995. doi: 10.1038/s41598-021-91497-5 (PMC8184817; doi:10.1038/s41598-021-91497-5)
Supplement: Supplementary file 1 — Supplementary Information. [file 41598_2021_91497_MOESM1_ESM.pdf]

Supplementary Information

Transmission of light signals from the light-oxygen-voltage core via the hydrophobic region of the β-sheet surface in aureochrome-1

Hiroto Nakajima, Itsuki Kobayashi, Yumiko Adachi and Osamu Hisatomi*

Department of Earth and Space Science, Graduate School of Science, Osaka University, Toyonaka, Osaka 560-0043, Japan. *e-mail: hisatomi@ess.sci.osaka-u.ac.jp

This supplementary information containing Figure S1–S10 and Table S1–S3

Figure S1. Spectral changes of the hinge and β-sheet mutants during dark regeneration
Figure S2. The $R_{H(app)}$ against protein concentrations of the hinge and β-sheet mutants
Figure S3. Correlation between R_H and apparent MW of the hinge and β-sheet mutants
Figure S4. EMSA data of the hinge and β-sheet mutants for dsApo with the target sequence
Figure S5. EMSA data of the hinge and β-sheet mutants for dsCpo without the target sequence
Figure S6. QCM data of the hinge and β-sheet mutants for dsApo
Figure S7. Spectral changes of the A'α mutants during dark regeneration
Figure S8. EMSA data of the A'α mutants for dsApo with the target sequence
Figure S9. QCM data of the A'α mutants for dsApo
Figure S10. Correlation between R_H and EC_{50} values of PZ mutants
Table S1. Sequences of oligonucleotides used in the present study
Table S2. Peak elution volumes of wtPZ and PZ mutants from SEC
Table S3. Peak elution volumes of the A' α mutants from SEC

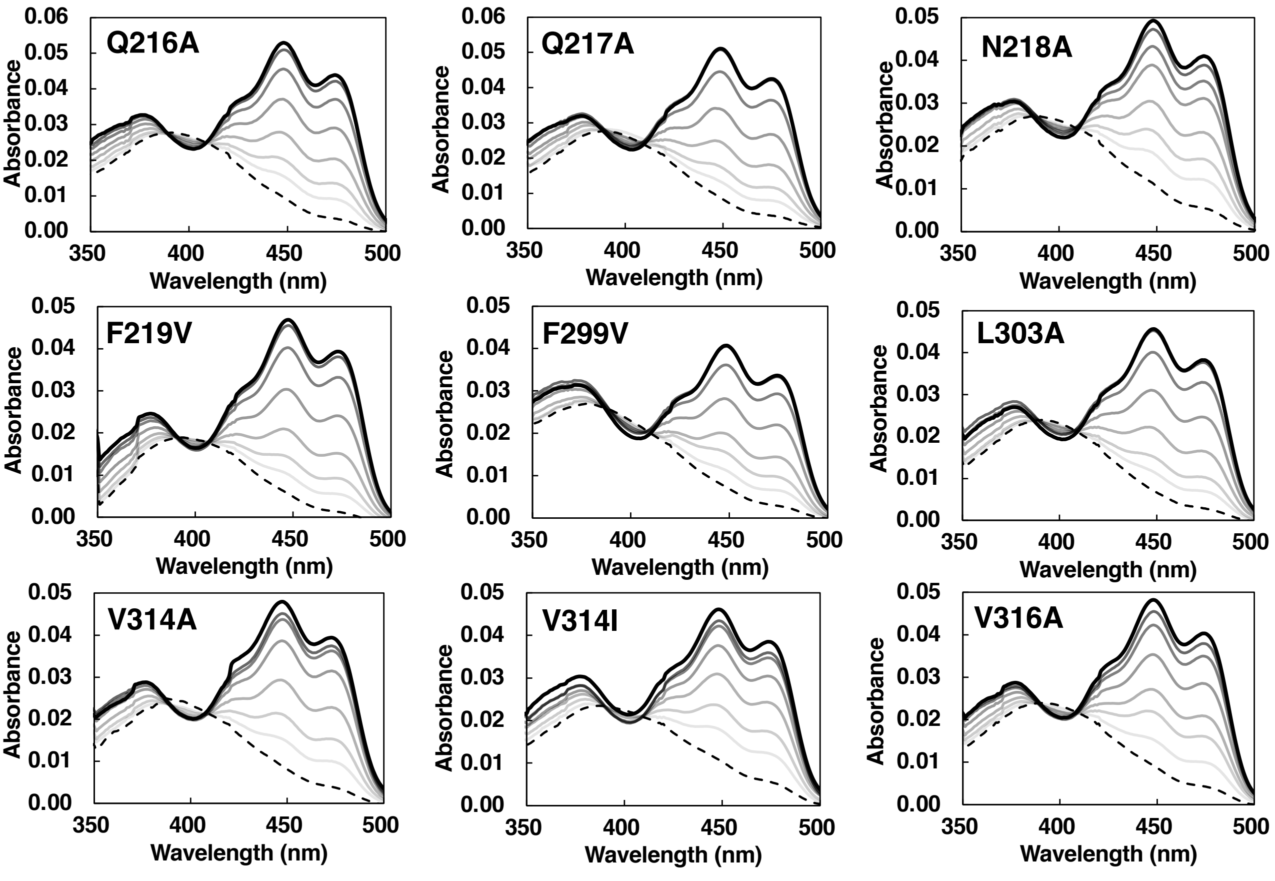

Figure S1. Spectral changes of the hinge and β-sheet mutants during dark regeneration. The spectra were measured in the initial dark state (solid black line), blue light immediately (dashed line), during dark regeneration (gray lines). Incubation times during dark regenerations are 2, 4, 8, 16, 32 and 64 min.

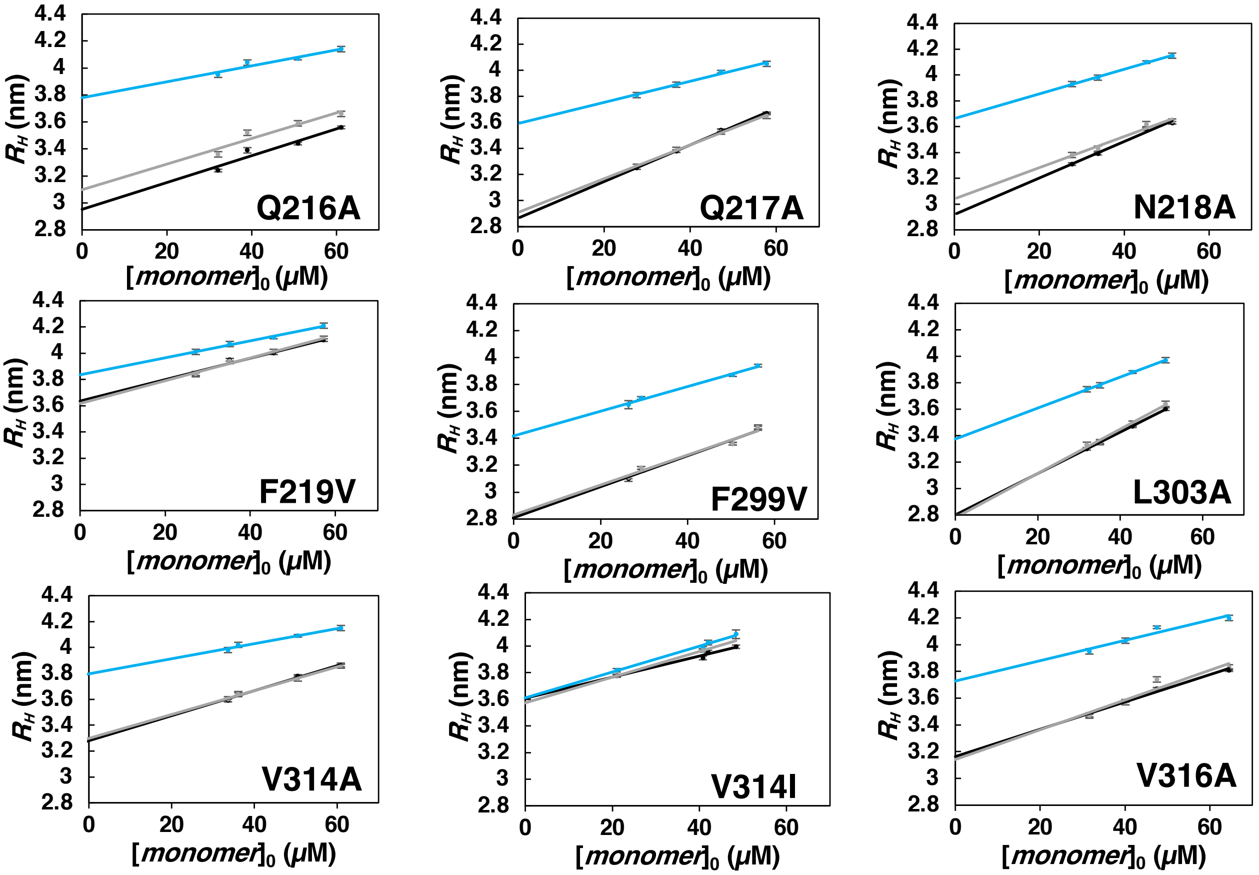

Figure S2. The $R_{H(app)}$ according to protein concentration relative to [*monomer*]$_0$ of the hinge and β-sheet mutants. Black, blue and gray symbols and lines represent the D, L and LD states, respectively. Measurements were performed more than 6 times for each concentration in the D, L and LD states.

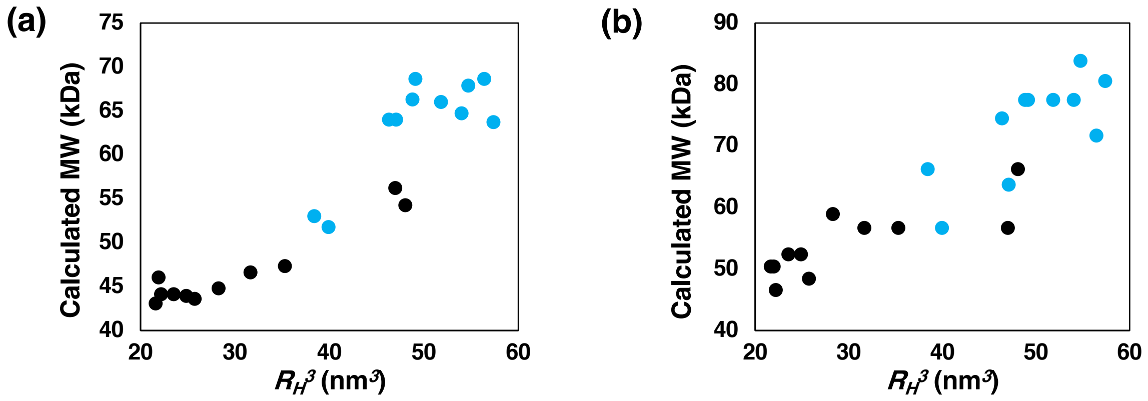

Figure S3. Correlation between R_H^3 and apparent MW of wtPZ and PZ mutants. MW were estimated from the peak elution volumes of SEC using the equation for globular proteins. Injected protein concentrations were (**a**) 8 µM and (**b**) 50 µM. Black and blue symbols represent data in the D and L states, respectively.

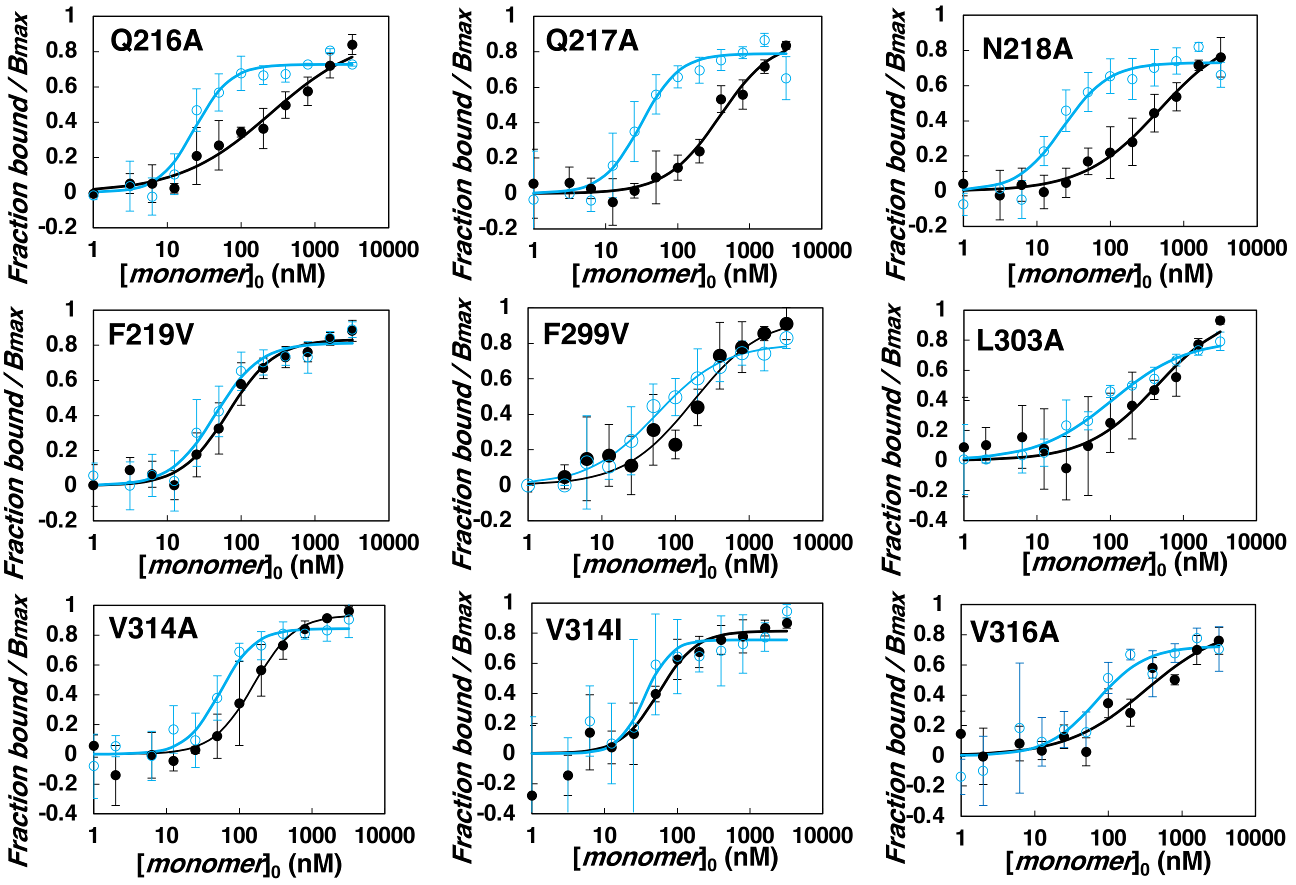

Figure S4. The normalized dsApo fractions bound to the hinge and β-sheet mutants plotted against applied protein concentrations $[monomer]_0$ with fitting curves, in the D (filled black circles and black lines) and L (open blue circles and blue lines) states. Measurements were performed more than 4 times each in the D and L state.

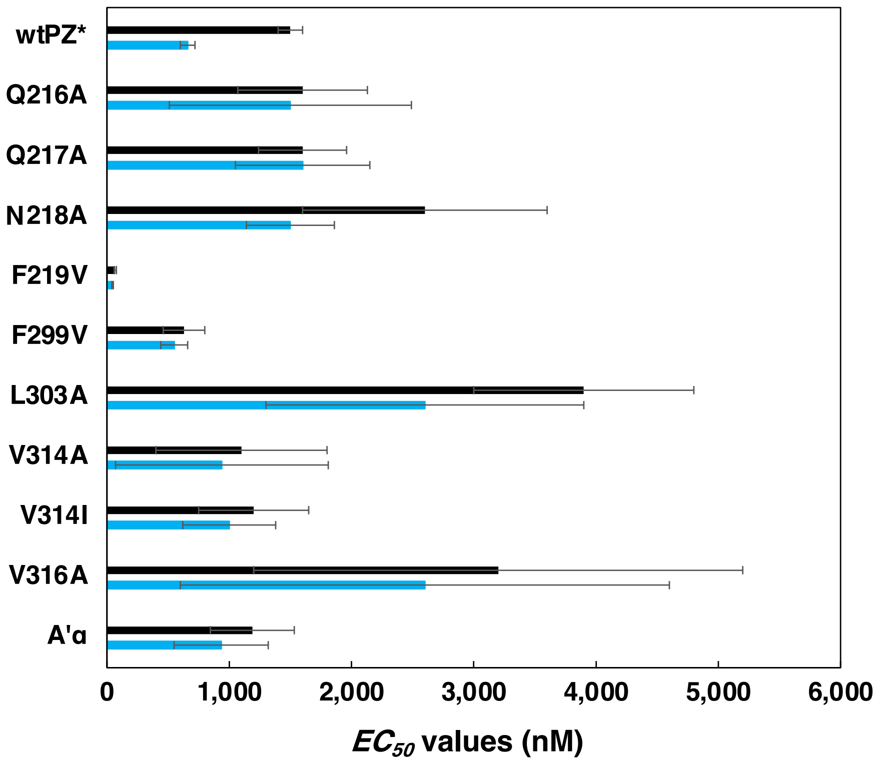

Figure S5. The EC_{50} values of wtPZ and PZ mutants for a control (nonspecific) palindromic oligonucleotide (dsCpo) according to EMSA in the D (black) and L (blue) states. Measurements were performed more than 3 times for each concentration in the D and L state. *Data for wtPZ are from a previous study[15].

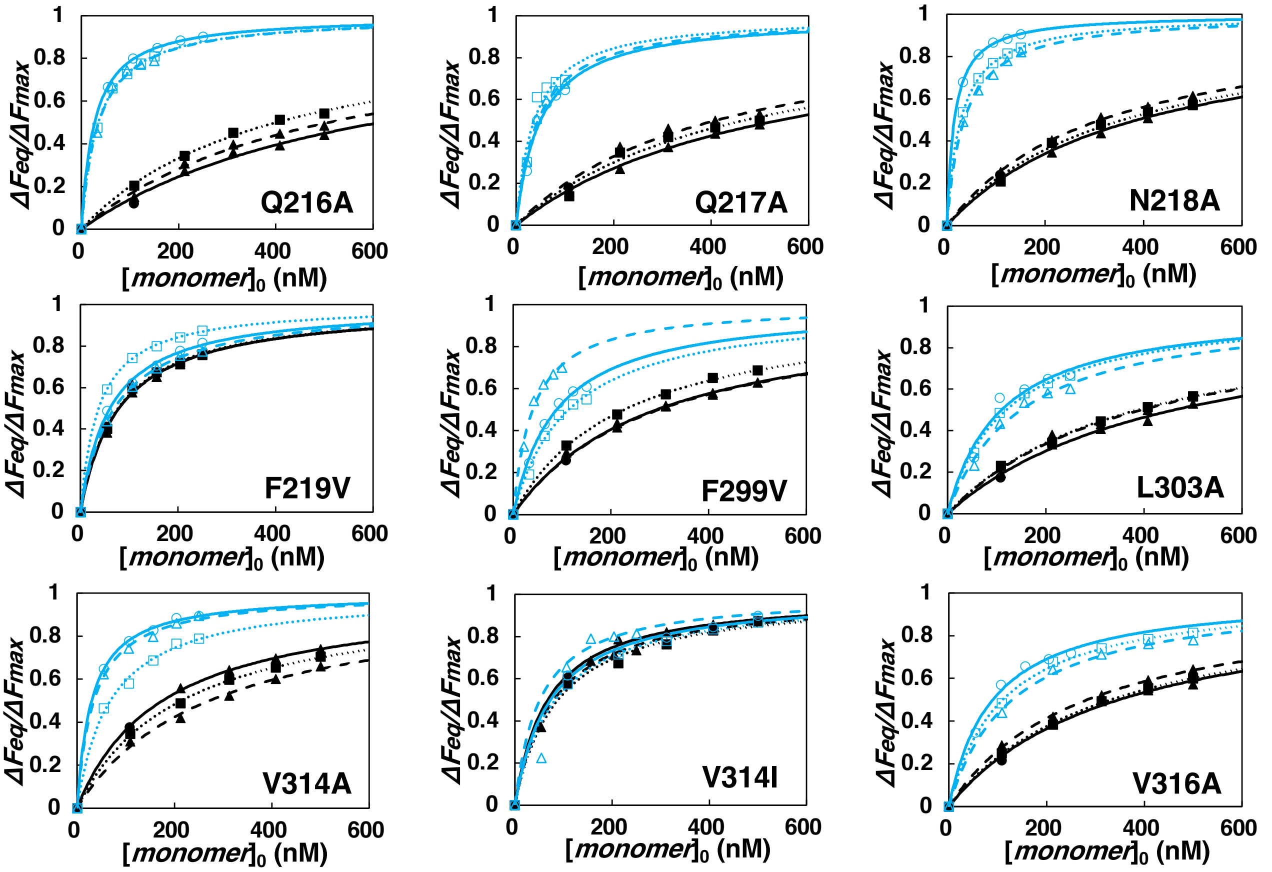

Figure S6. Saturation binding behaviors ($\Delta F/\Delta F_{max}$) against $[monomer]_0$ of the hinge and β-sheet mutants. Filled black circles and black lines indicate in the D state, and open blue circles and blue lines indicate in the L state. Measurements were performed more than 3 times each in the D and L state.

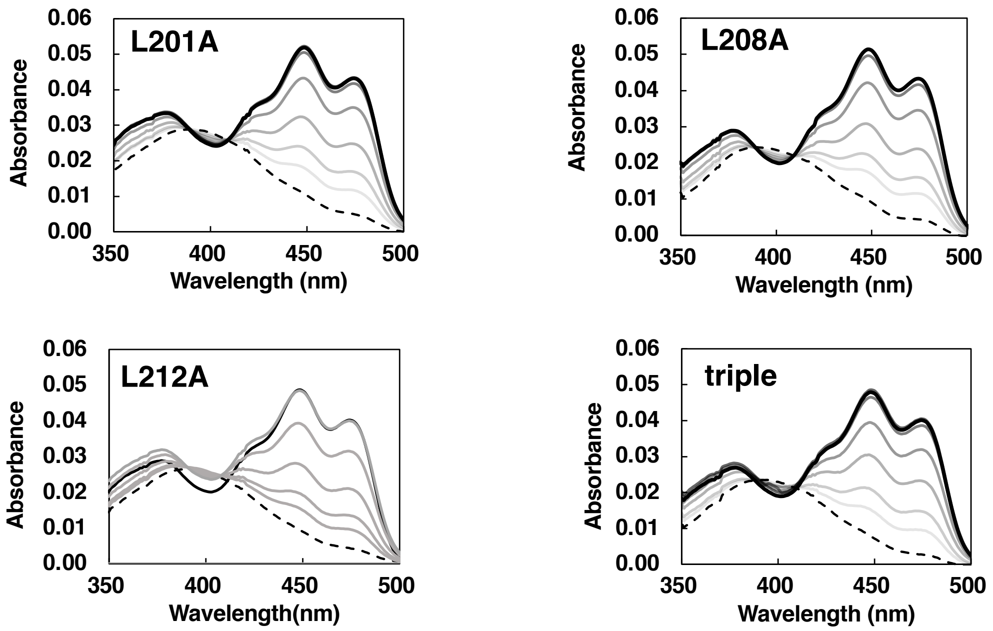

Figure S7. Spectral changes of the A'α mutants during dark regeneration. The spectra were measured in the initial dark state (solid black line), blue light immediately (dashed line), during dark regeneration (gray lines). Incubation times during dark regenerations are 2, 4, 8, 16, 32 and 64 min.

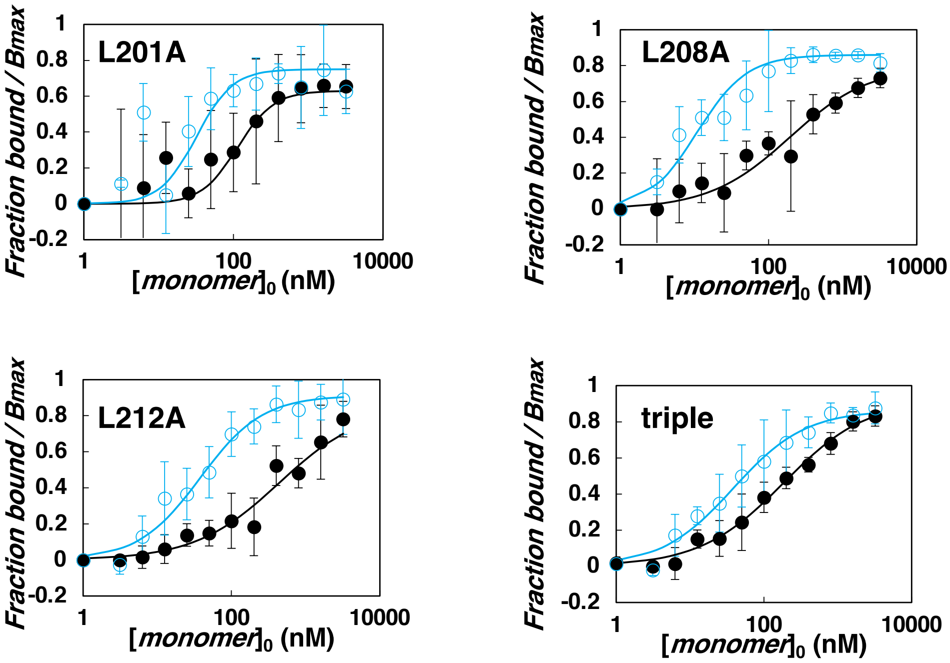

Figure S8. The normalized dsApo fractions bound to the A'α mutants plotted against applied protein concentrations $[monomer]_0$ with fitting curves, in the D (filled black circles and black lines) and L (open blue circles and blue lines) states. Measurements were performed more than 4 times each in the D and L state.

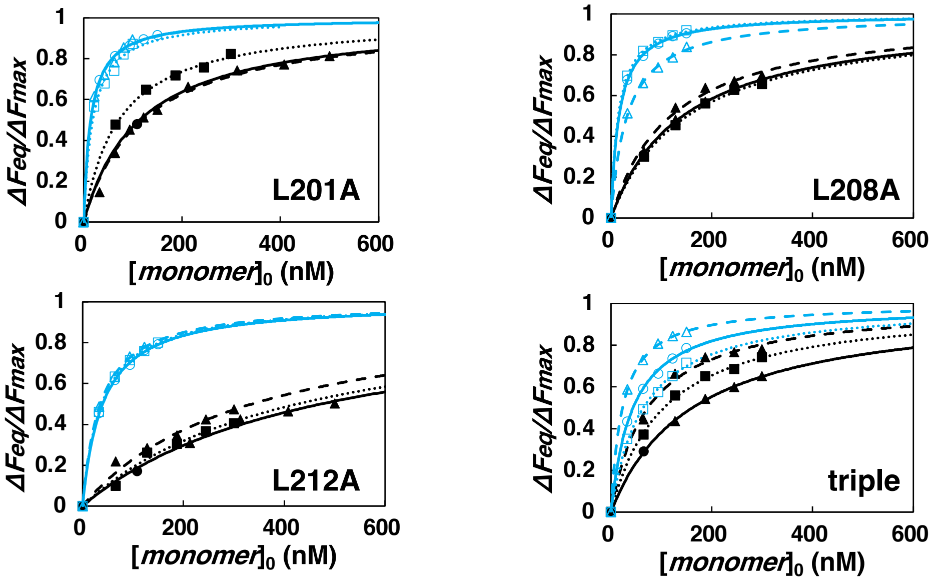

Figure S9. Saturation binding behaviors ($\Delta F/\Delta F_{max}$) against $[monomer]_0$ of the A'α mutants. Filled black circles and black lines indicate in the D state, and open blue circles and blue lines indicate in the L state. Measurements were performed more than 3 times each in the D and L state.

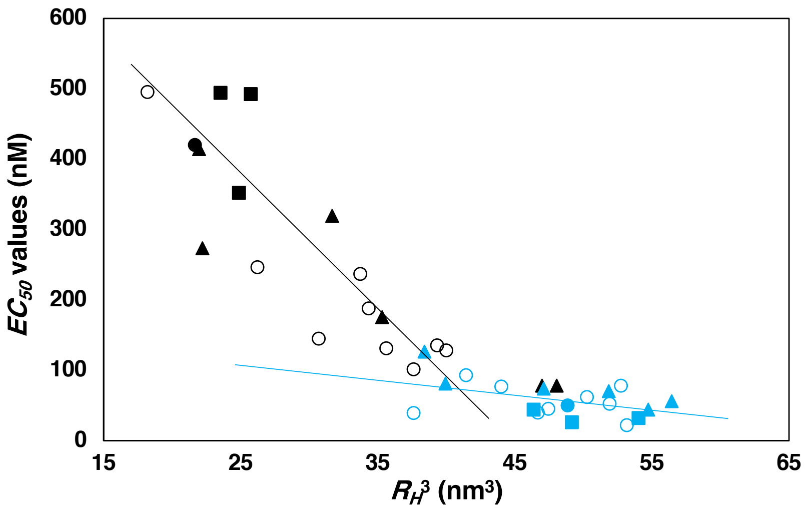

Figure S10. Correlation between EC_{50} and R_H^3 values of wtPZ and PZ mutants. Filled circles, squares and triangles represent wtPZ, β-sheet mutants and hinge mutants, respectively, and black and blue symbols represent in the D and L states, respectively. Open circles are data for the F298 and Q317 mutants[17].

Table S1. Oligonucleotide sequences used in this study.

PZ_Q216A-F	5' – ATGGCCGCTCAGAACTTCGTGATTACC – 3'
PZ_Q216A-R	5' – GTTCTGAGCGGCCATTTGCAGGGCTTT – 3'
PZ_Q217A-F	5' – GCCCAGGCTAACTTCGTGATTACCGAT – 3'
PZ_Q217A-R	5' – GAAGTTAGCCTGGGCCATTTGCAGGGC – 3'
PZ_N218A-F	5' – CCAGCAGGCCTTCGTGATTACCGATGC – 3'
PZ_N218A-R	5' – CACGAAGGCCTGCTGGGCCATTTGCAG – 3'
PZ_F219V-F	5' – GCAGAACGTCGTGATTACCGATGCAA – 3'
PZ_F219V-R	5' – ATCACGACGTTCTGCTGGGCCATTTG – 3'
PZ_F299V-F	5' – CCTGTTTGTCGTGGCTGGCCTGCGCG – 3'
PZ_F299V-R	5' – GCCACGACAAACAGGTTCCAAAAAGT – 3'
PZ_L303A-F	5' – GCTGGCGCCCGCGATTCCAAAGGTAAC – 3'
PZ_L303A-R	5' – ATCGCGGGCGCCAGCCACGAAAAACAG – 3'
PZ_V314A-F	5' – AACTACGCCGGCGTTCAGAGTAAAGTC – 3'
PZ_V314A-R	5' – AACGCCGGCGTAGTTAACGATGTTACC – 3'
PZ_V314I-F	5' – AACTACATTGGCGTTCAGAGTAAAGTC – 3'
PZ_V314I-R	5' – AACGCCAATGTAGTTAACGATGTTACC – 3'
PZ_V316A-F	5' – GTGGGCGCTCAGAGTAAAGTCTCCGA – 3'
PZ_V316A-R	5' – ACTCTGAGCGCCCACGTAGTTAACGA – 3'
PZ_L201A-F	5' - CGCATTGCTGAAGATCCGGACTATAGC - 3'
PZ_L201A-R	5' – ATCTTCAGCAATGCGATTGGCTTTGGA - 3'
PZ_L208A-F	5' – TATAGCGCTGTGAAAGCCCTGCAAATG - 3'
PZ_L208A-R	5' – TTTCACAGCGCTATAGTCCGGATCTTC - 3'
PZ_L212A-F	5' – AAAGCCGCTCAAATGGCCCAGCAGAAC - 3'
PZ_L212A-R	5' – CATTTGAGCGGCTTTCACCAGGCTATA - 3'
PZ_L208A/L212A-R	5' – CATTTGAGCGGCTTTCACAGCGCTATA - 3'
Apo	5' – GCTGTCTGACGTCAGACAGC – 3'
Cpo	5' – GCTGTGCAGATCTGCACAGC – 3'

The underline indicates the target sequence of *Vf*AUREO1[11].

Table S2. Peak elution volumes of wtPZ and PZ mutants

	8μM		50μM	
	D (mL)	L (mL)	D (mL)	L (mL)
PZ	9.9	8.7	9.5	8.4
Q216A	9.9	8.9	9.6	8.4
Q217A	9.8	8.9	9.4	8.5
N218A	9.8	8.7	9.4	8.4
F219V	9.3	8.7	8.8	8.6
F299V	9.8	9.4	9.7	9.2
L303A	9.7	9.4	9.5	8.8
V314A	9.7	8.7	9.2	8.2
V314I	9.2	8.9	8.7	8.7
V316A	9.7	8.8	9.2	8.4

Table S3. Peak elution volumes of A'α mutants

	8μM		50μM	
	D (mL)	L (mL)	D (mL)	L (mL)
L201A	9.8	8.6	9.0	8.3
L208A	9.8	8.7	9.0	8.3
L212A	9.9	8.9	9.4	8.4
triple	9.8	8.7	9.0	8.3
